# Erratum: Ruthenium-cobalt nanoalloys encapsulated in nitrogen-doped graphene as active electrocatalysts for producing hydrogen in alkaline media

**DOI:** 10.1038/ncomms16029

**Published:** 2017-06-20

**Authors:** Jianwei Su, Yang Yang, Guoliang Xia, Jitang Chen, Peng Jiang, Qianwang Chen

Nature Communications 8: Article number: 14969; DOI: 10.1038/ncomms14969 (2017); Published: 04 25 2017; Updated: 06 20 2017

In the original version of this Article, which was received on 04 November 2016, the received date was incorrectly given as 04 November 2017. This has now been corrected in both the PDF and HTML versions of the Article.

