# Corrigendum: Ruthenium-cobalt nanoalloys encapsulated in nitrogen-doped graphene as active electrocatalysts for producing hydrogen in alkaline media

**DOI:** 10.1038/ncomms16028

**Published:** 2017-06-20

**Authors:** Jianwei Su, Yang Yang, Guoliang Xia, Jitang Chen, Peng Jiang, Qianwang Chen

Nature Communications 8: Article number: 14969; DOI: 10.1038/ncomms14969 (2017); Published: 04 25 2017; Updated: 06 20 2017

The legend to Fig. 8 of this Article contains a typographical error. The first sentence of this legend should read ‘(a) Co and (b) Ru_3_Co models.’

The Results section contains two typographical errors. The penultimate sentence of the first paragraph of the section ‘Electrochemical characterization for HER catalysis’ should read ‘It was also found that S-4 with lower Ru content (3.58 wt.% Ru in RuCo@NC catalyst, obtained from ICP) showed better activity than the etched counterpart with higher Ru content (17.7 wt.% Ru in RuCo alloy, obtained from ICP), which was in good agreement with the above-mentioned trend of overpotentials.’ In the same section, ‘Especially, the S-4 catalyst exhibited an ASA*C_S_ of 1.242 mA cm^−2^’ should read ‘Especially, the S-4 catalyst exhibited an ASA*C_S_ of 1.242*C_S_ mA cm^−2^’.

The legend to Supplementary Fig. 14 incorrectly states that the Δj values plotted in h) correspond to those at 0.15 V *vs* RHE. The Δ*j* values in fact correspond to those at 0.1 V *vs* RHE.

