# Supplementary material for: Ruthenium-cobalt nanoalloys encapsulated in nitrogen-doped graphene as active electrocatalysts for producing hydrogen in alkaline media
Source: Nat Commun. 2017 Apr 25;8:14969. doi: 10.1038/ncomms14969 (PMC5413983; doi:10.1038/ncomms14969)
Supplement: Supplementary Information — Supplementary Figures, Supplementary Tables, Supplementary Note and Supplementary References [file ncomms14969-s1.pdf]

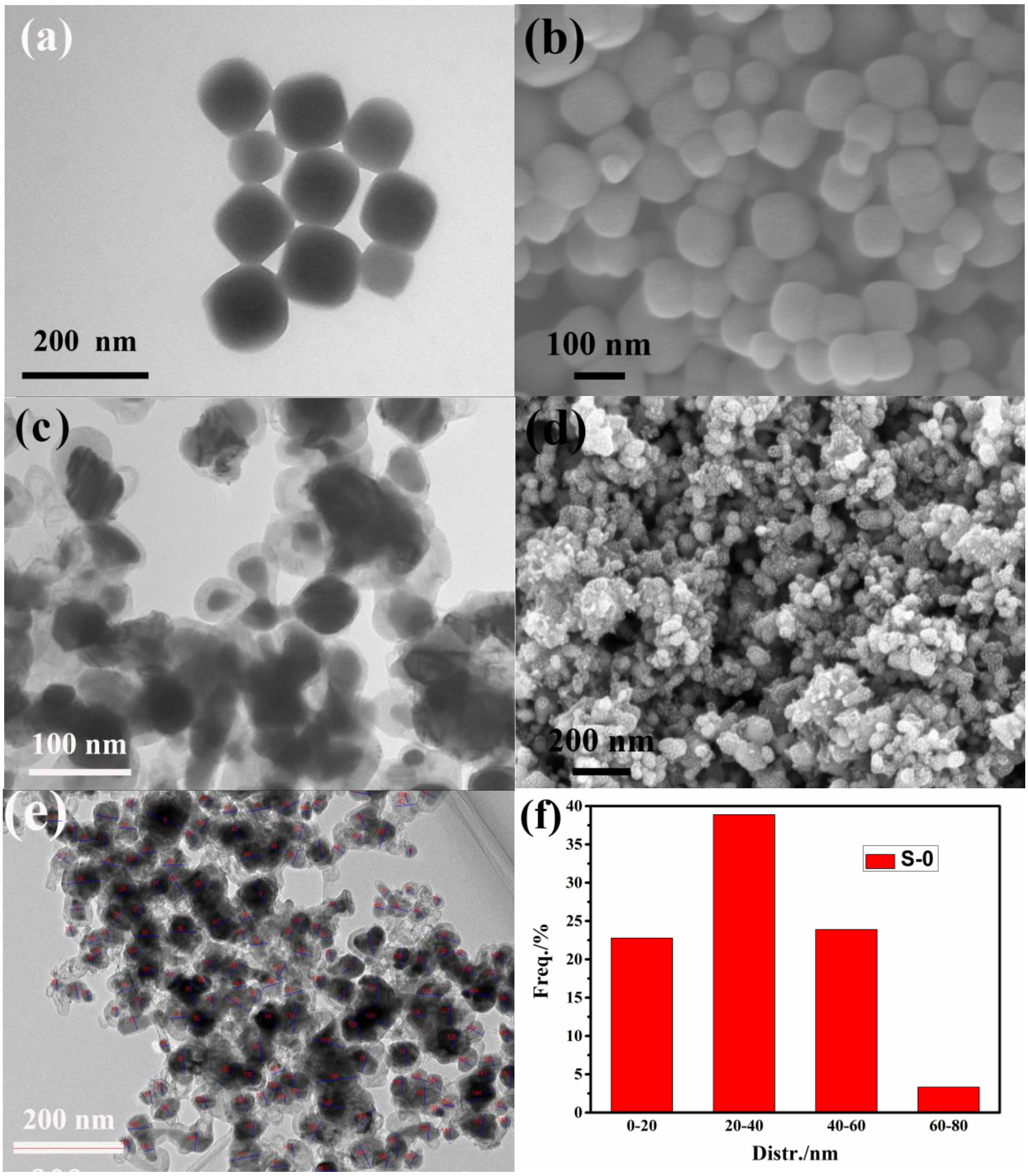

Supplementary Figure 1. SEM and TEM characterization of S-0-MOF and S-0. (a, b) TEM and SEM images of S-0-MOF, (c, d) TEM and SEM images of the corresponding annealed sample S-0, (e,f) Statistical analysis of the particle sizes of Co metal in S-0.

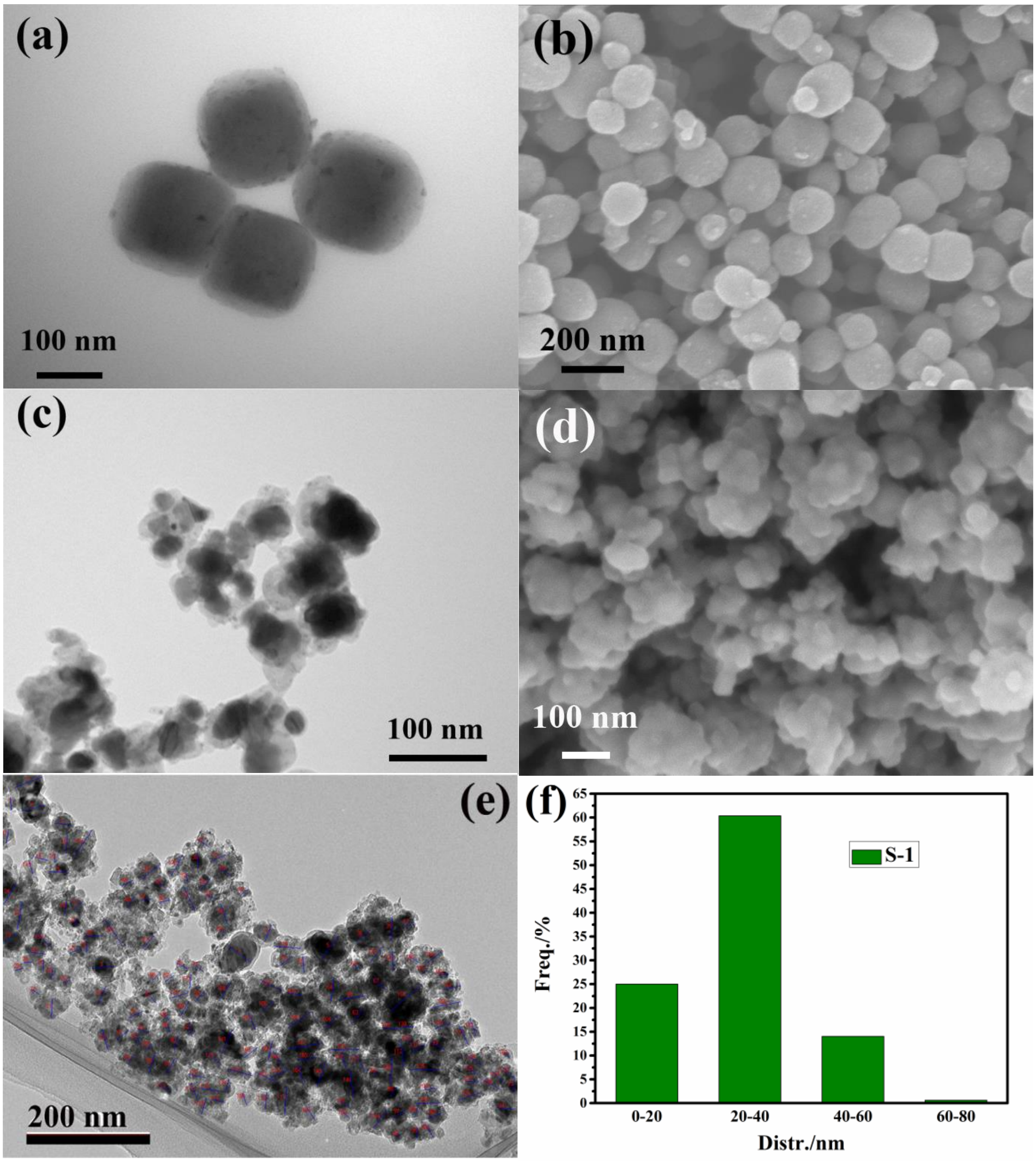

Supplementary Figure 2. SEM and TEM characterization of S-1-MOF and S-1. (a, b) TEM and SEM images of S-1-MOF, (c, d) TEM and SEM images of the corresponding annealed sample S-1, (e,f) Statistical analysis of the particle sizes of RuCo alloy in S-1..

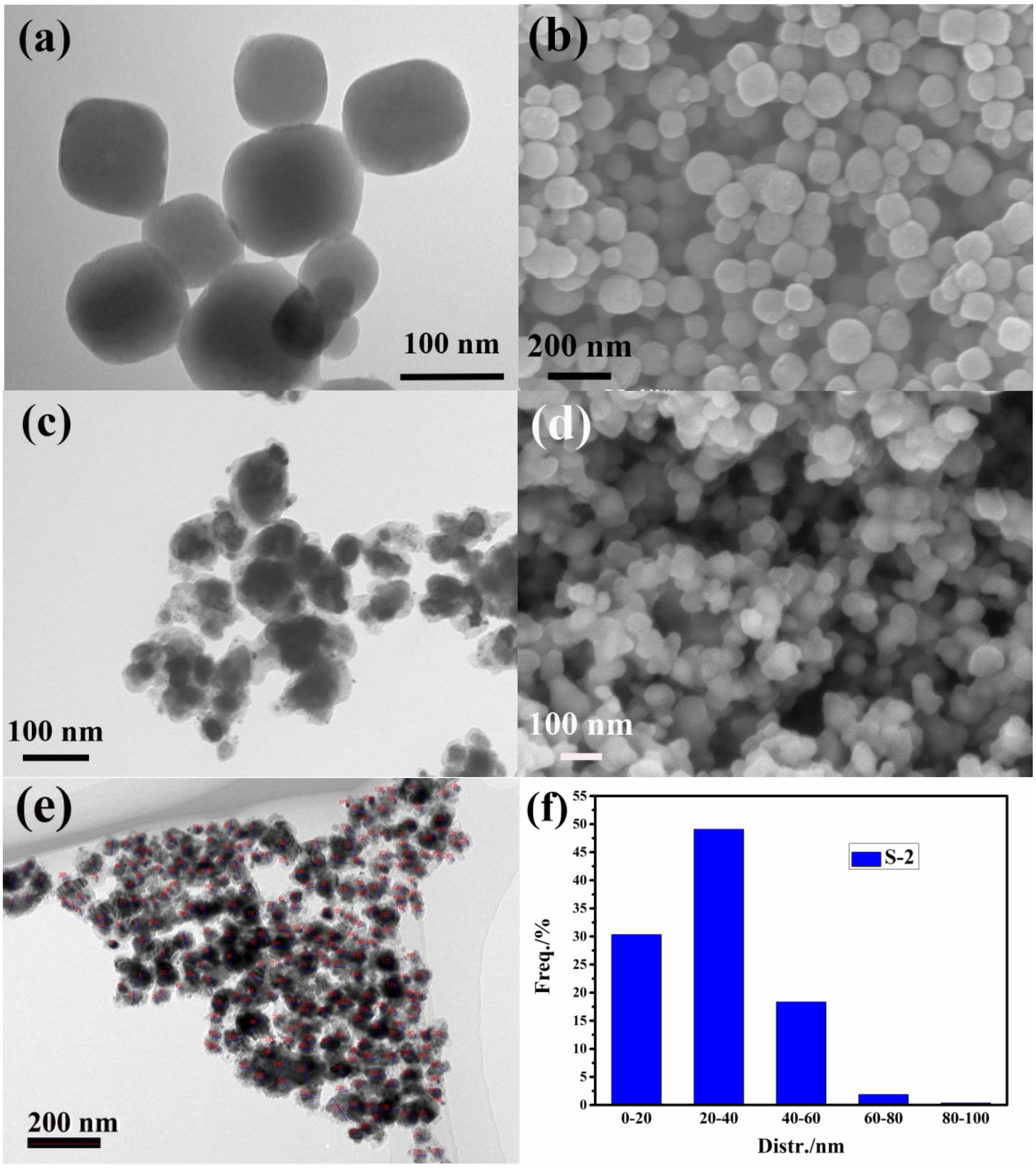

Supplementary Figure 3. SEM and TEM characterization of S-2-MOF and S-2. (a, b) TEM and SEM images of S-2-MOF, (c, d) TEM and SEM images of the corresponding annealed sample S-2, (e,f) Statistical analysis of the particle sizes of RuCo alloy in S-2.

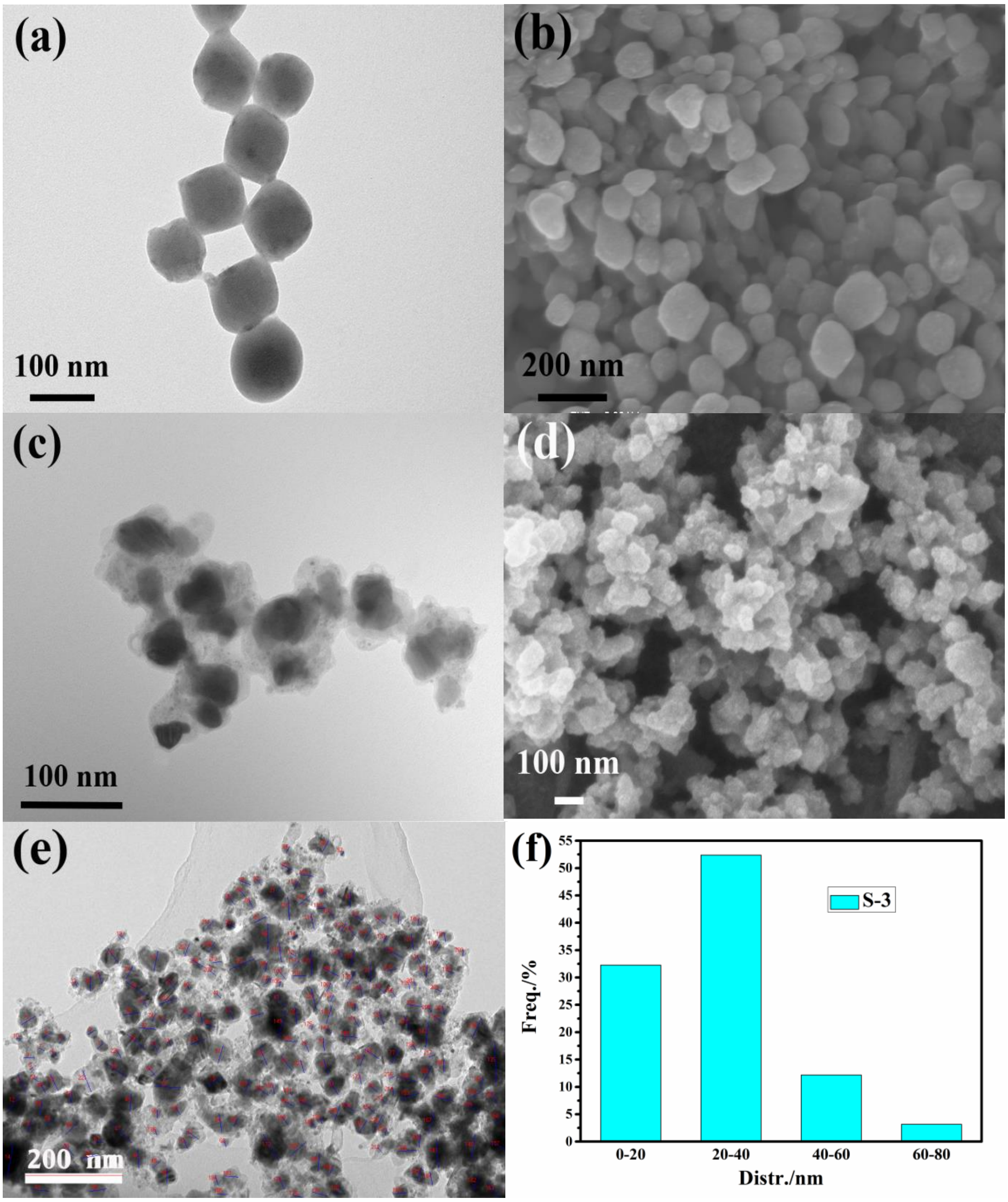

Supplementary Figure 4. SEM and TEM characterization of S-3-MOF and S-3. (a, b) TEM and SEM images of S-3-MOF, (c, d) TEM and SEM images of the corresponding annealed sample S-3, (e,f) Statistical analysis of the particle sizes of RuCo alloy in S-3.

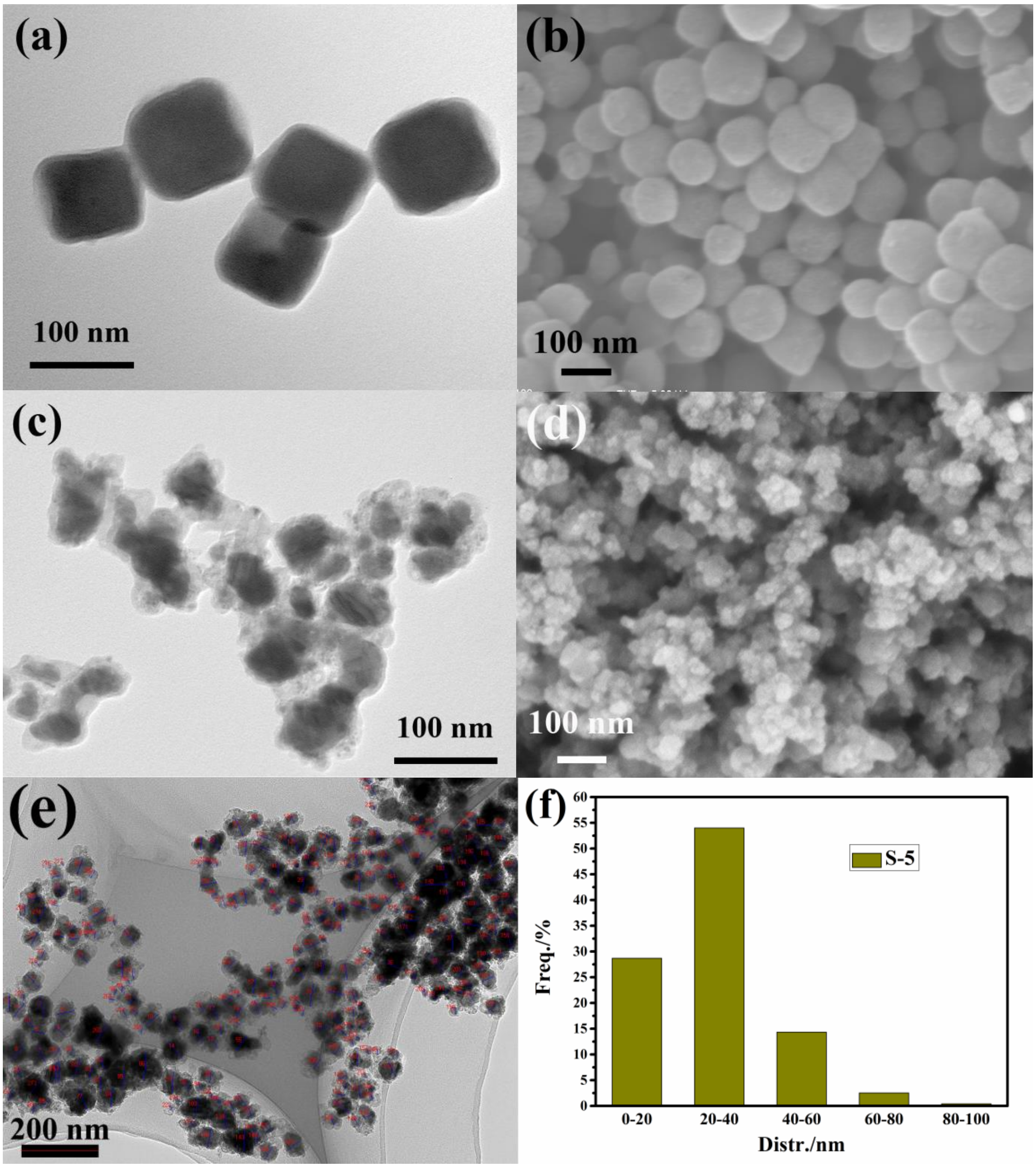

Supplementary Figure 5. SEM and TEM characterization of S-5-MOF and S-5. (a, b) TEM and SEM images of S-5-MOF, (c, d) TEM and SEM images of the corresponding annealed sample S-5, (e,f) Statistical analysis of the particle sizes of RuCo alloy in S-5.

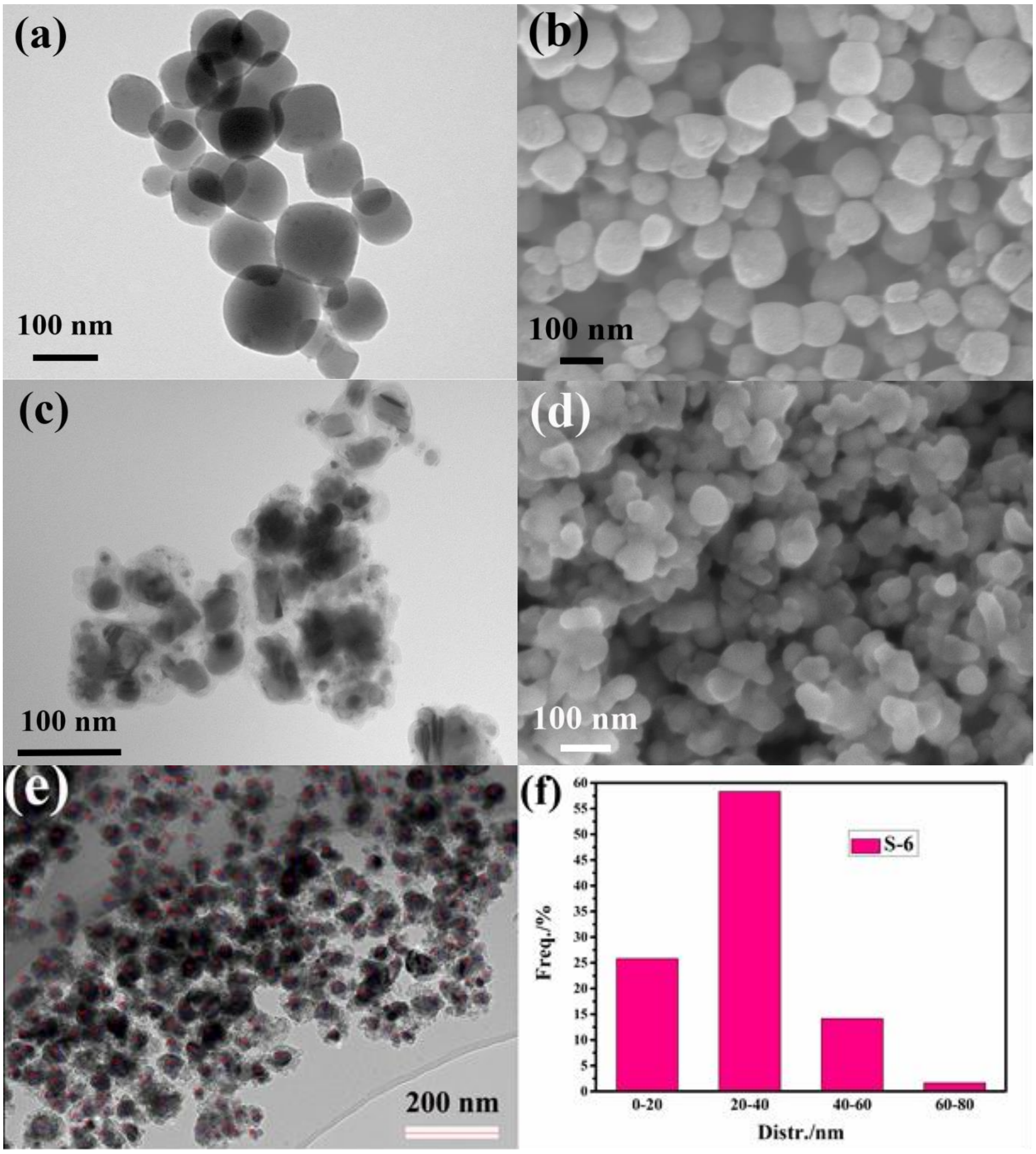

Supplementary Figure 6. SEM and TEM characterization of S-6-MOF and S-6. (a, b) TEM and SEM images of S-6-MOF, (c, d) TEM and SEM images of the corresponding annealed sample S-6, (e,f) Statistical analysis of the particle sizes of RuCo alloy in S-6.

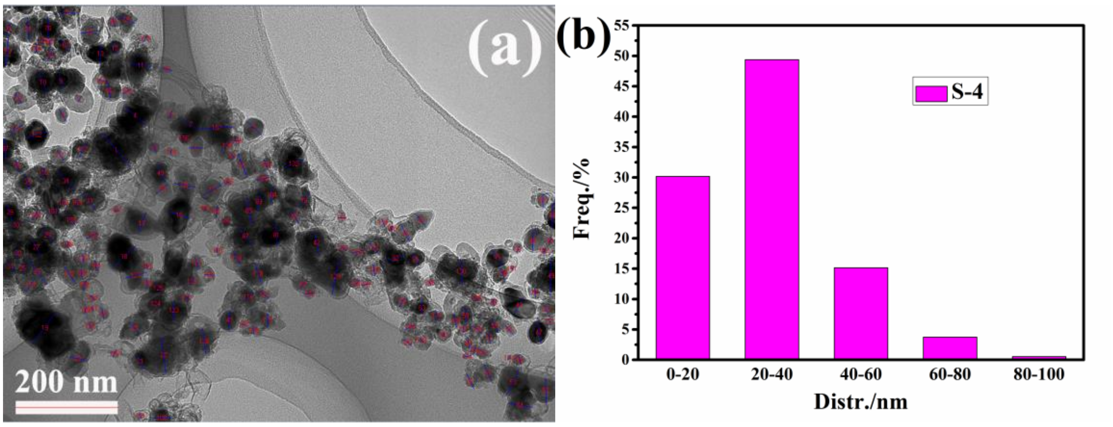

Supplementary Figure 7. (a,b) Statistical analysis of the particle sizes of RuCo alloy in S-4.

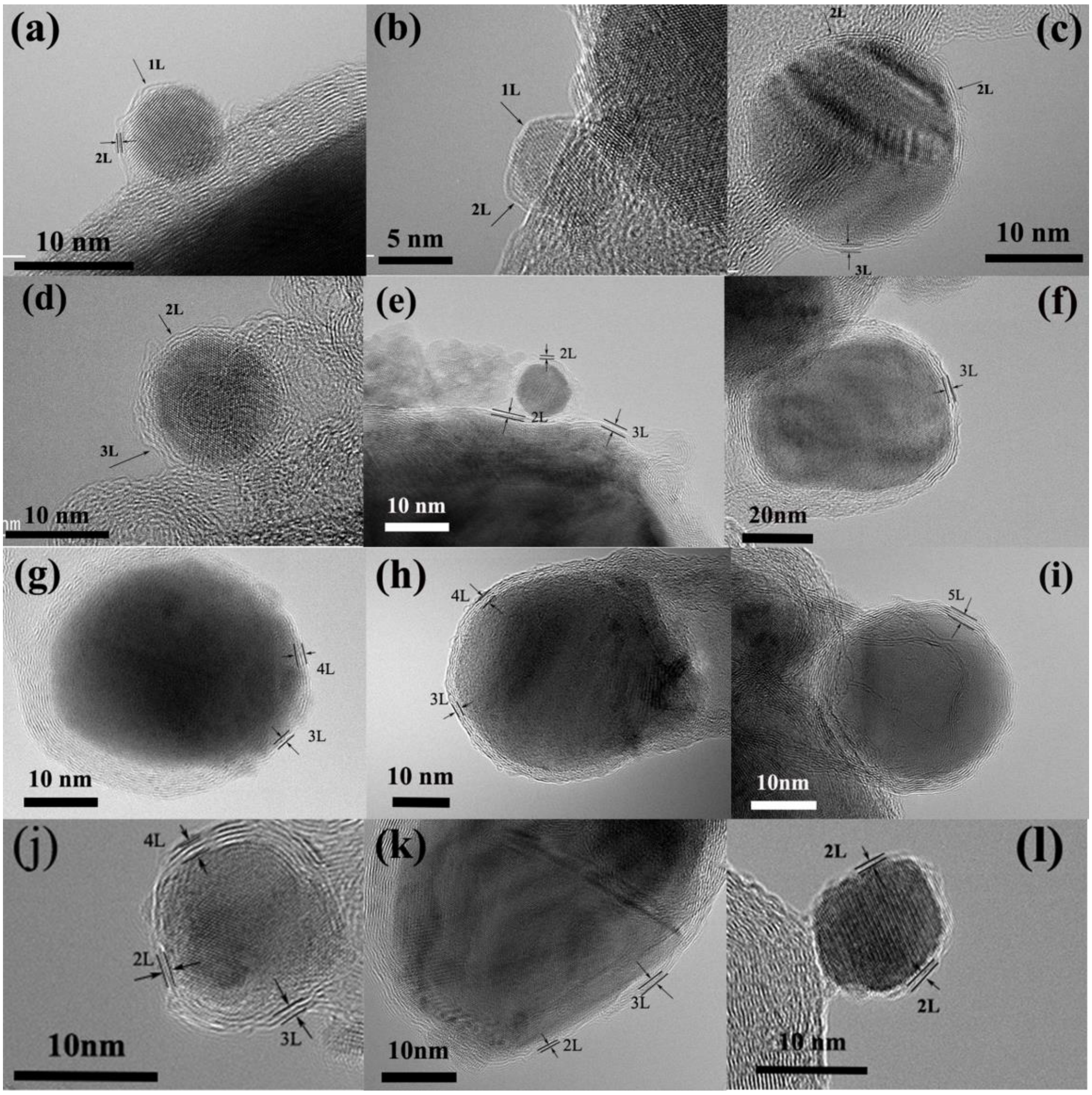

Supplementary Figure 8. High resolution transmission electron microscopy (HRTEM) of the S-4.

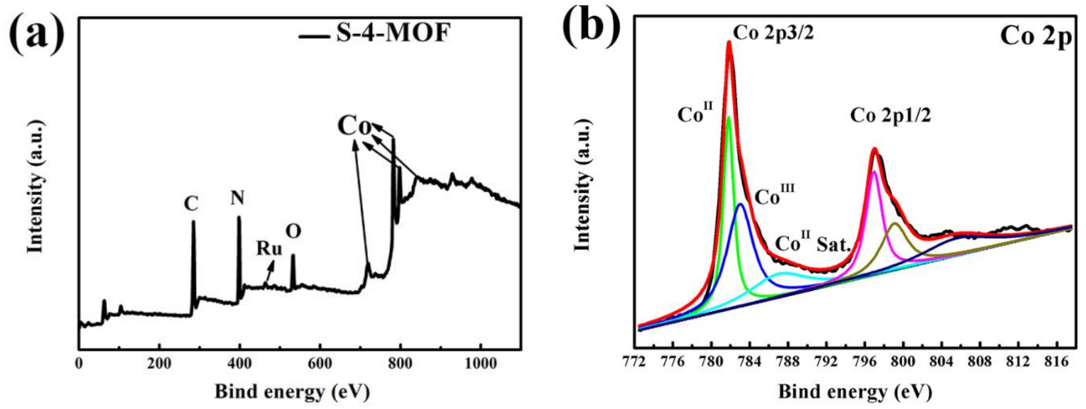

Supplementary Figure 9. XPS Characterization of S-4-MOF. (a) The XPS spectra of S-4-MOF, (b) XPS result of the Co2p spectrum enlarged in Supplementary Figure 9a.

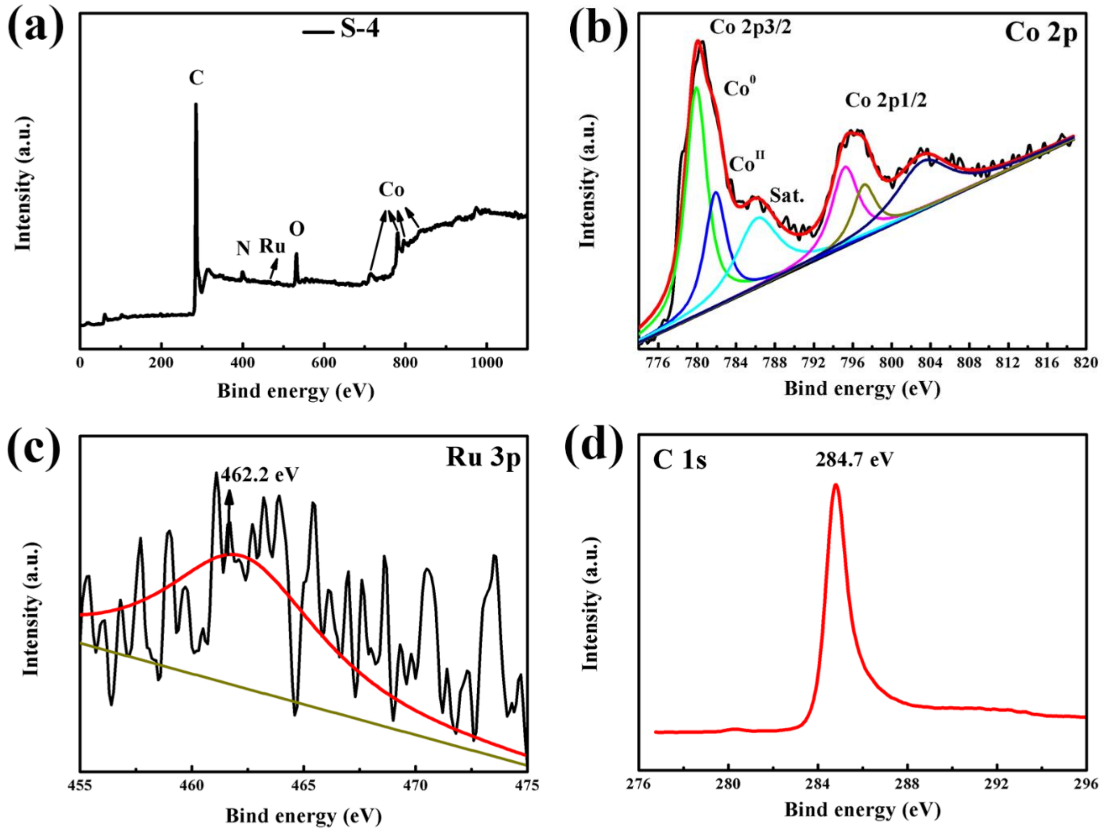

Supplementary Figure 10. XPS Characterization of S-4. (a) The XPS spectra of S-4, (b-d) The XPS result of the Co2p, Ru3p and C1s spectrum enlarged in Supplementary Figure 10a.

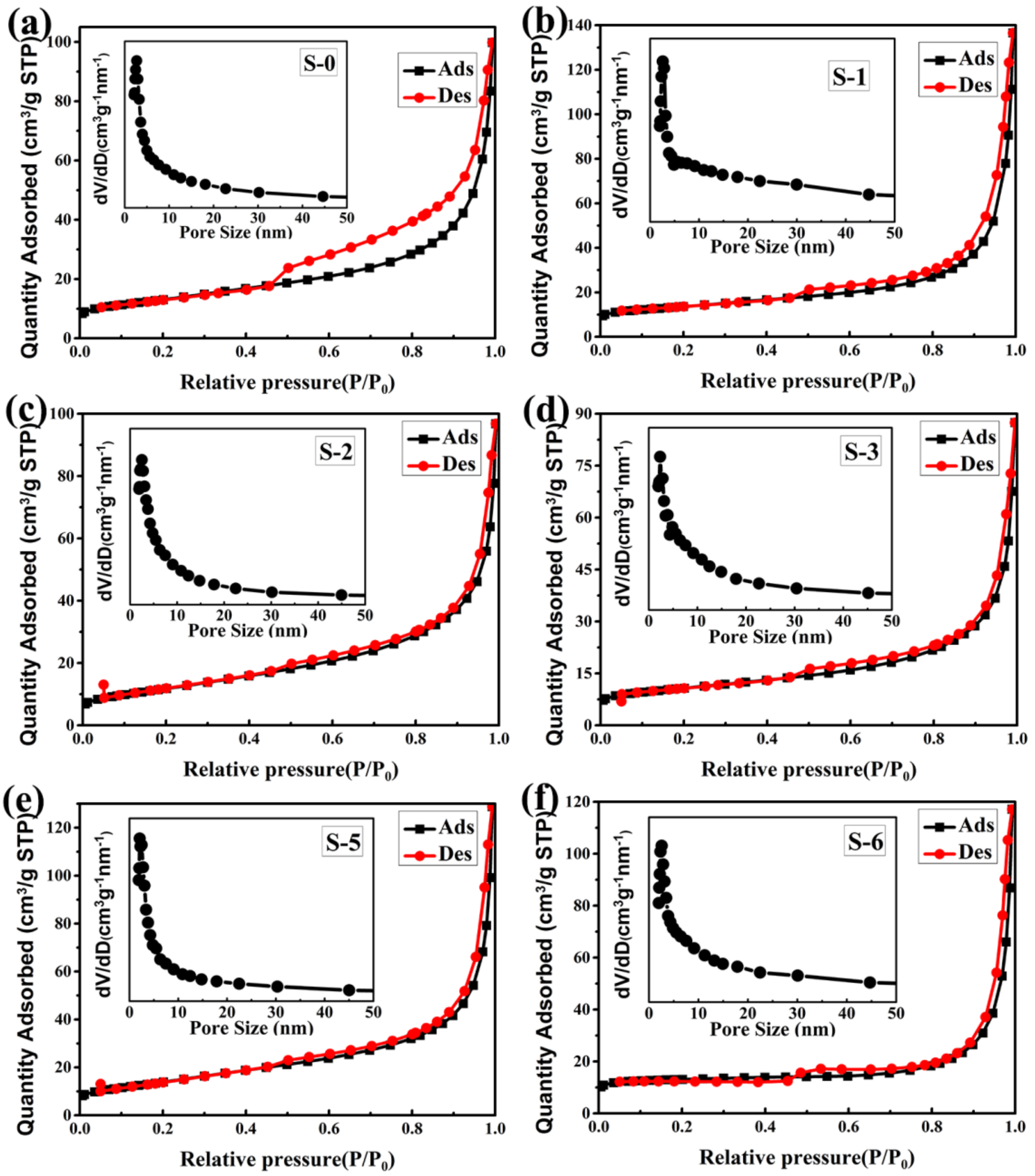

Supplementary Figure 11. N_2 adsorption–desorption isotherm and pore size distribution plot (inset) of S-0, S-1, S-2, S-3, S-5 and S-6.

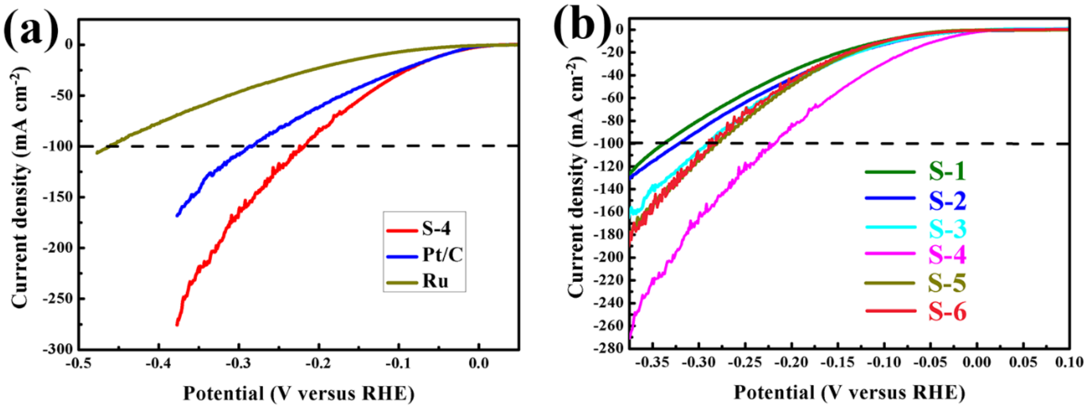

Supplementary Figure 12. Electrocatalytic HER performance test of catalysts at the larger overpotential. (a) HER polarization curves of S-4, Ru and Pt/C at the bigger overpotential, (b) HER polarization curves of CoRu@NC samples at the larger overpotential.

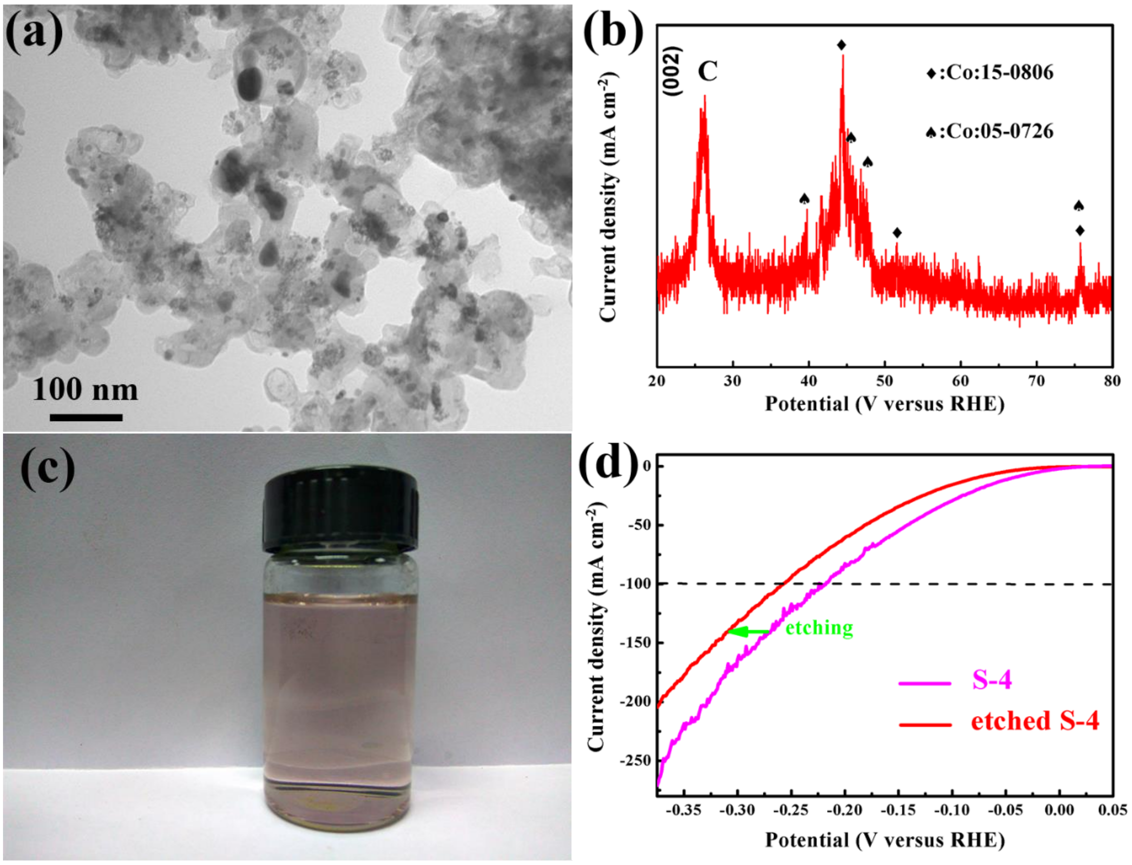

Supplementary Figure 13. Characterization of the etched S-4 .(a) The TEM image of etched S-4 by 1M HCl, (b) The XRD pattern of the etched S-4, (c) The photo of the pink solution after etching, (d) HER polarization curve of the etched S-4 reaching the bigger overpotential.

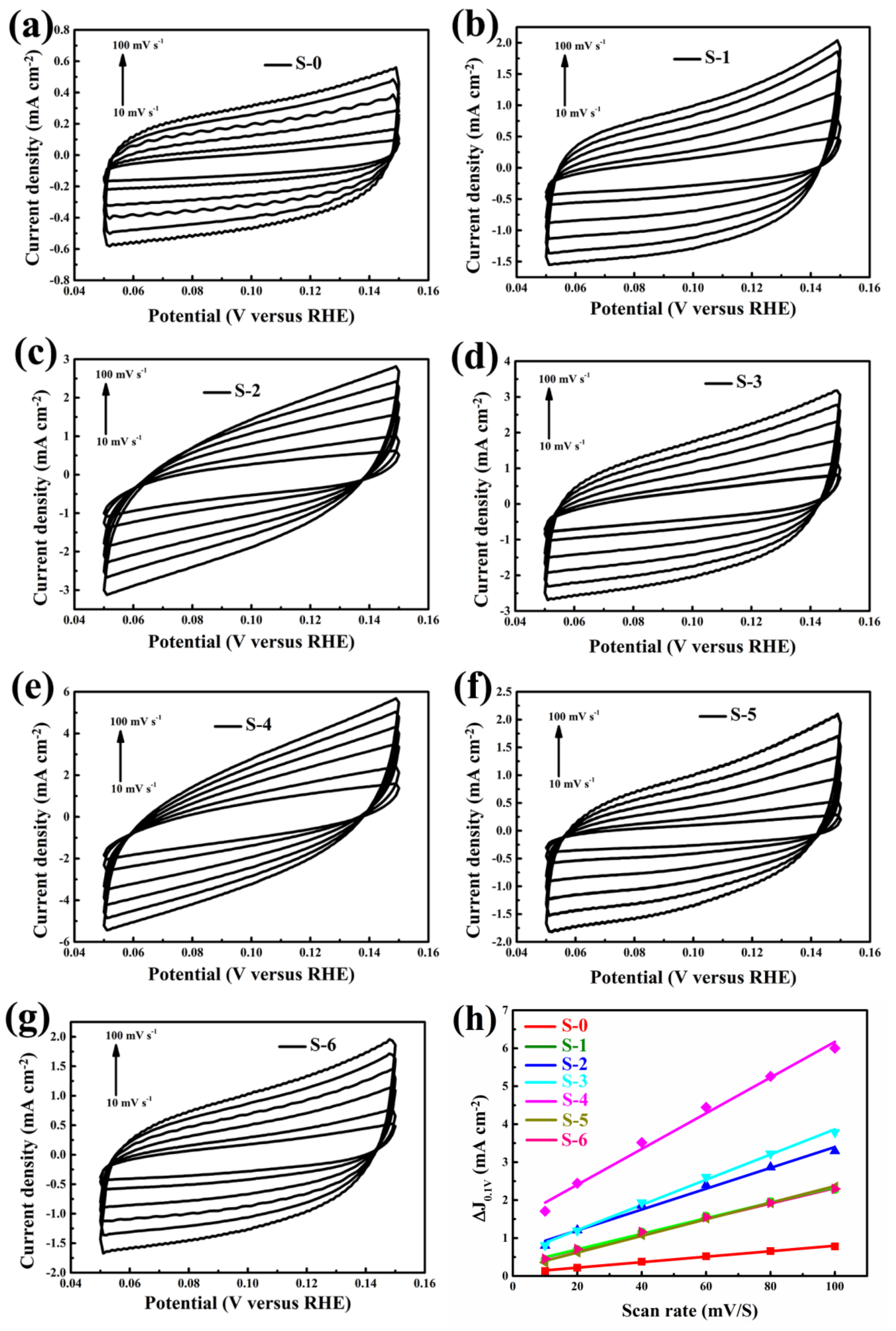

Supplementary Figure 14. Electrochemically active surface area measurements. (a-h) CV curves measured within the range of 0.05 to 0.15 V *vs* RHE with scan rate from 10 to 100 mV s^{-1} and corresponding △j at 0.15 V vs RHE *vs* scan rates plots of S-0, S-1, S-2, S-3, S-4, S-5 and S-6.

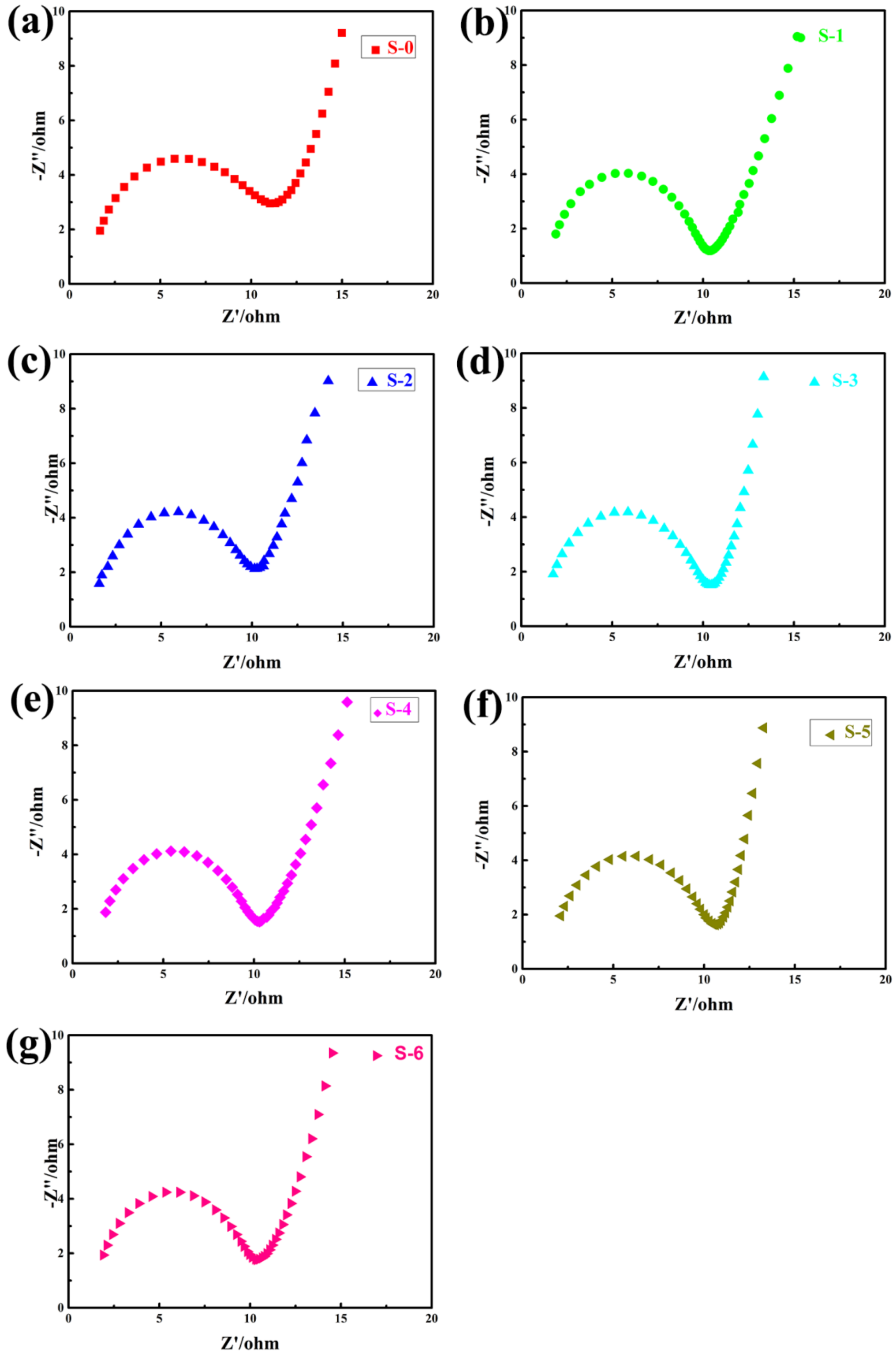

Supplementary Figure 15. Electrochemical impedance spectroscopy (EIS) Nyquist plots for S-0, S-1, S-2, S-3, S-4, S-5 and S-6 collected in frequency range of 1–10^5 Hz.

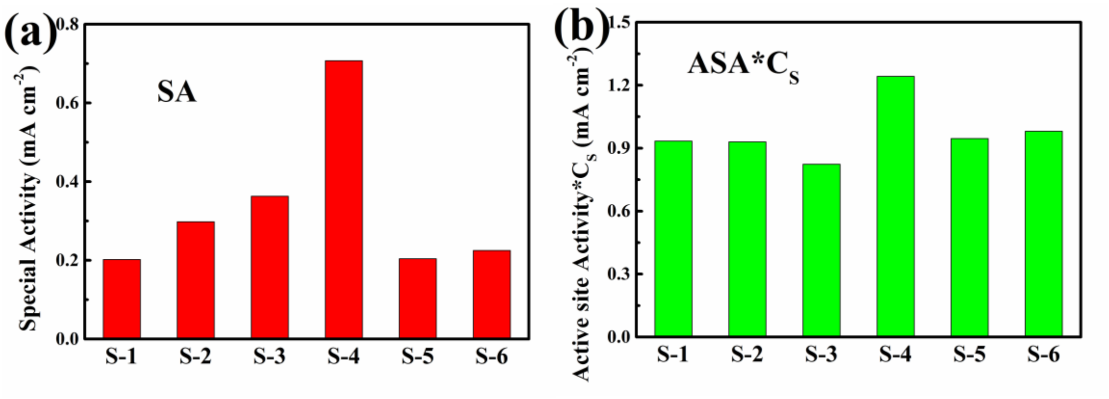

Supplementary Figure 16. Activity normalization. Activity normalization of (a) special activity (SA) and (b) active site activity (ASA) taking into account of the surface area and active site concentration respecitively at overpotential of 100 mV.

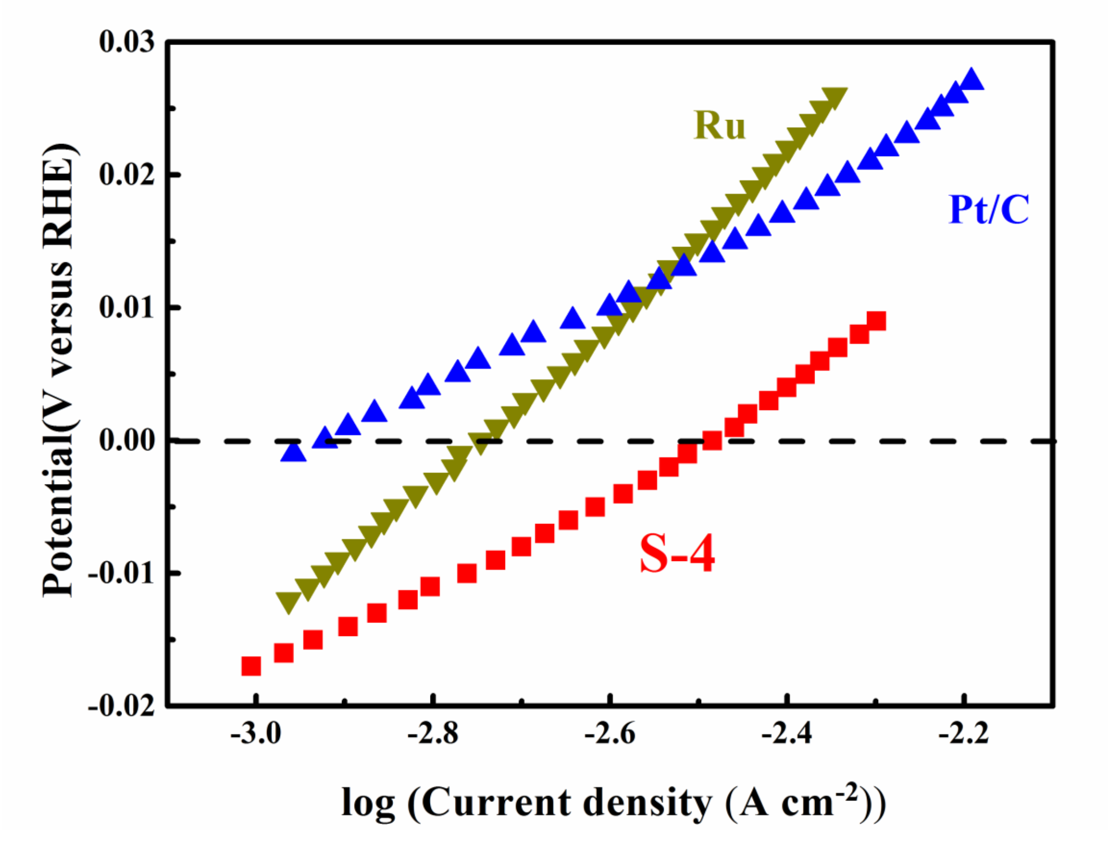

Supplementary Figure 17. The exchange current density of S-4, Ru and Pt/C.

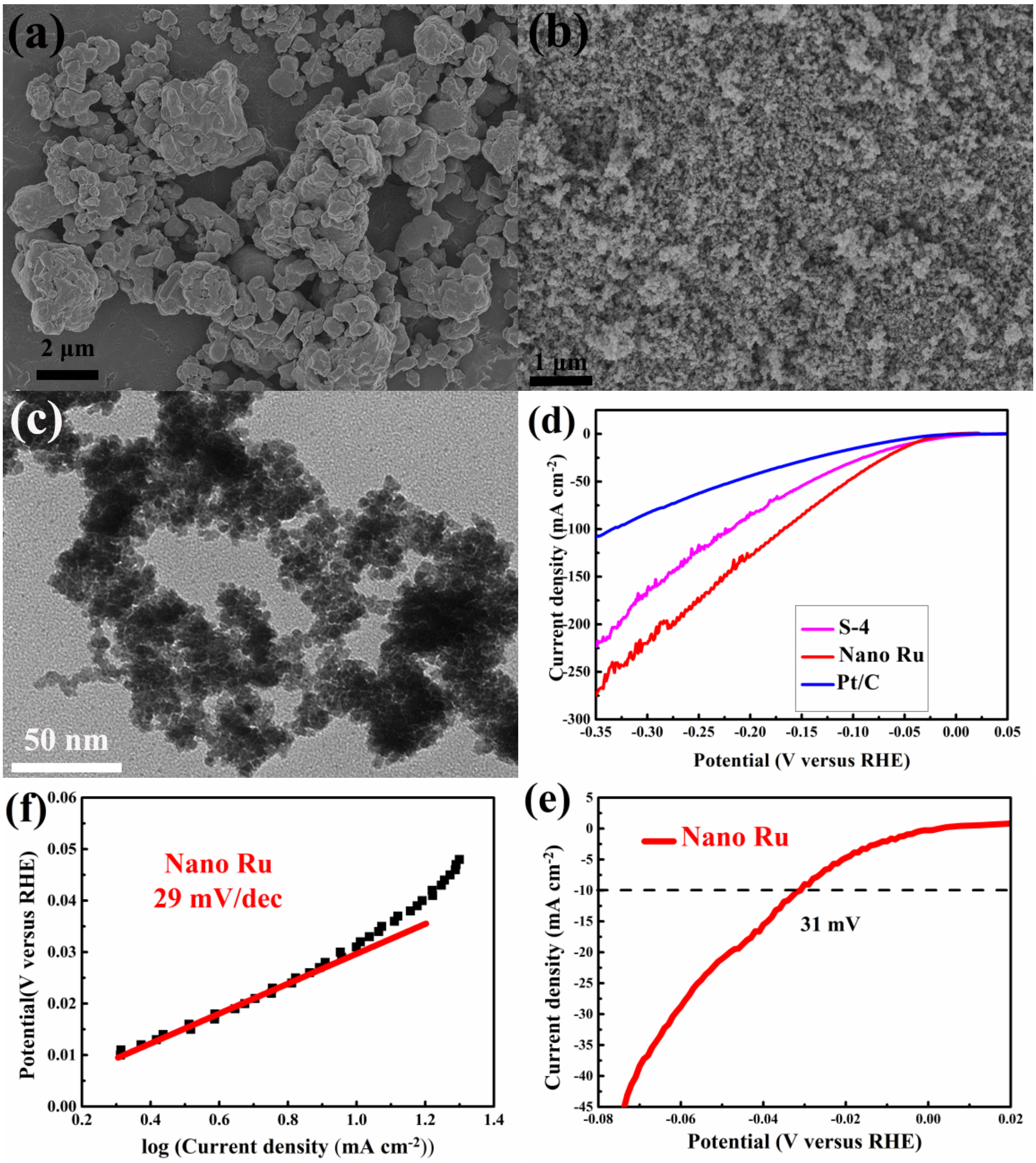

Supplementary Figure 18 Characterization of nano Ru. (a) The FESEM image of the commercial Ru powder catalysts. (b and c) The FESEM and TEM images of our obtained nano-sized Ru. (d-f) HER polarization curves and the Tafel plot of Nano Ru.

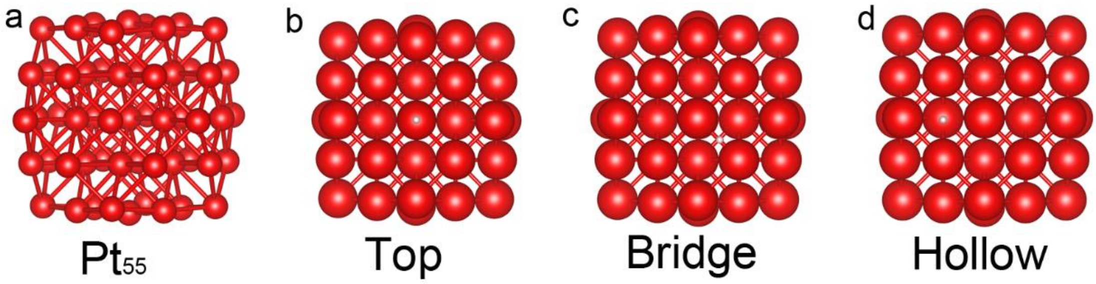

Supplementary Figure 19. Cluster model of Pt_{55}. (a) Pt_{55} cluster model from side view, (b-d) H* adsorbed on top, bridge, and hollow sites on cluster from top view, respectively.

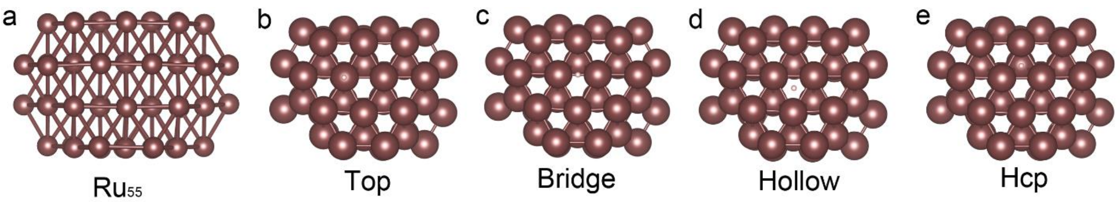

Supplementary Figure 20. Cluster model of Ru_{55}. (a) Ru_{55} cluster model from side view, (b-e) H* adsorbed on top, bridge, hollow and hcp sites on cluster from top view, respectively.

Supplementary Table 1. Mean particle size, specific surface area and double layer capacitance of various catalysts.

Catalyst	S-0	S-1	S-2	S-3	S-4	S-5	S-6
Mean particle size(nm)	30.87	28.09	28.75	28.21	28.38	29.94	28.28
Specific surface area(m^2 g^{-1})	46.49	47.41	42.87	37.68	41.37	50.77	44.27
Double layer capacitance(mF/cm^2)	3.11	10.27	13.73	16.61	23.55	10.95	10.16

Supplementary Table 2. Calculated best free energies ΔG_{H^*} of various models and adsorption sites.

Models	graphene	N-gra	Co	Ru1Co	Ru2Co	Ru3Co	Ru55(bridge)	Pt55(top)
$\triangle G_{H^*}$ (eV)	2.64	0.52	0.49	0.47	0.43	0.31	-0.03	-0.30

Supplementary Table 3. Total number of transferred electrons from metal to graphene based on Bader charge analysis.

Models	Co	Ru_1Co	Ru_2Co	Ru_3Co
Transferred electron(e^-)	5.81	5.84	5.83	5.91

Supplementary Table 4. Calculated ΔGH* of different adsorption sites on Pt55 and Ru55.

Models/Sites	Top	Bridge	Hollow	HCP
Pt_{55}	-0.30	-0.37	-0.37	-
Ru_{55}	-0.09	0.03	-0.09	-0.07

Supplementary Note 1

Calculation details: We perform DFT calculations using the Vienna Ab Initio Simulation Package (VASP),[1, 2] the generalized gradient approximation (GGA) of Perdew−Becke−Ernzerhof (PBE) is used for the exchange-correlation functional.[3] A graphitic carbon cage C_{240} encapsulated 55 metal atoms was used as the model of graphene encapsulated alloys, which performed well in previous study.[4, 5, 6] The cut-off energies for plane waves is 400 eV, providing a convergence of 10^{-4} eV in total energy and 0.05 eV/Å in Hellmann Feynman force on each atom. The hydrogen binding energy $\triangle E_H$ was calculated by $\triangle E_H = E_{H\text{-}slab} - E_{slab} - 1/2\ E_{H2}$. The free energies at 298.15 K were obtained using $\triangle G=\triangle E_H+\triangle ZPE\text{-}T\triangle S$ according to previous work where $\triangle ZPE\text{-}T\triangle S$=0.37eV for model of graphene encapsulated metal.[6, 7, 8] ΔE_H is the hydrogen binding energy, ΔZPE, ΔS and U are the zero point energy changes and entropy changes, respectively.

Supplementary References

1. Hoshino K & Shimojo F. Ab *initio* molecular dynamics for expanded and compressed liquid alkali metals. *J. Phys.: Condens. Matter* **8**, 9315-9319 (1996).
2. Kresse G. & Joubert D. From ultrasoft pseudopotentials to the projector augmented-wave method. *Phys. Rev. B* **59**, 1758-1775 (1999).
3. Perdew J. P., Burke K. & Ernzerhof M. Generalized Gradient Approximation Made Simple. *Phys. Rev. Lett.* **77**, 3865-3868 (1996).
4. Cui X. J., Ren P. J., Deng D. H., Deng J. & Bao X. H. Single layer graphene encapsulating non-precious metals as high-performance electrocatalysts for water oxidation. *Energ. Environ. Sci.* **9**, 123-129 (2016).
5. Deng J., Ren P. J., Deng D. H. & Bao X. H. Enhanced electron penetration through an ultrathin graphene layer for highly efficient catalysis of the hydrogen evolution reaction. *Angew. Chem. Int. Ed.* **54**, 2100-2104 (2015).
6. Deng J., Ren P. J., Deng D. H., Yu L., Yang F. & Bao X. H. Highly active and durable non-precious-metal catalysts encapsulated in carbon nanotubes for hydrogen evolution reaction. *Energ. Environ. Sci.* **7**, 1919-1923 (2014).
7. Zheng Y*. et al.* Hydrogen evolution by a metal-free electrocatalyst. *Nat. Commun.* **5**, 3783 (2014).
8. Norskov J. K. *et al.* Trends in the exchange current for hydrogen evolution. *J. Electrochem. Soc.* **152**, J23-J26 (2005).
